# Delayed diagnosis of homocystinuria presenting with coronavirus disease 2019 in a 17-year-old boy

**DOI:** 10.1590/0037-8682-0143-2022

**Published:** 2022-09-19

**Authors:** Nurhayat Yakut, Behzat Tuzun, Nurcan Ucuncu Ergun

**Affiliations:** 1 Basaksehir Cam and Sakura City Hospital, Department of Pediatrics, Division of Pediatric Infectious Diseases, Istanbul, Turkey.; 2 Basaksehir Cam and Sakura City Hospital, Department of Pediatric Cardiovascular Surgery, Istanbul, Turkey.; 3 Basaksehir Cam and Sakura City Hospital, Department of Pediatrics, Division of Pediatric Nutrition and Metabolism, Istanbul, Turkey.

**Keywords:** Homocystinuria, COVID-19, Thrombosis

## Abstract

Homocystinuria is a treatable autosomal recessive inherited disorder. This condition may cause life-threatening complications such as thromboembolic events. Coronavirus disease 2019 (COVID-19) is associated with an increased risk of venous thromboembolic events. Here, we report a case of late diagnosis of homocystinuria presenting with deep venous thrombosis and COVID-19. This study highlights a sustained high index of suspicion for homocystinuria to prevent severe thromboembolic complications.

## INTRODUCTION

By February 2022, the coronavirus disease 2019 (COVID-19) pandemic had affected almost 400 million people worldwide, with more than 2,800,000 new cases and 5,750,000 deaths^1^. Although previous reports demonstrated that pediatric patients had a milder clinical course and outcomes, complications such as venous thromboembolism have been reported in critically ill COVID-19 patients. Inflammatory response and damaged coagulation function may have contributed to thromboembolic events^2^ ^,^ ^3^. Homocystinuria is an autosomal recessive disorder caused by a deficiency in cystathionine beta-synthase enzyme activity. If untreated, this disorder may cause severe complications such as thromboembolic events^4^. Herein, we report a case of homocystinuria presenting with venous thrombosis and COVID-19 in a 17-year-old boy. 

## CASE REPORT

A 17-year-old boy was admitted to the emergency room with complaints of fever, cough, leg pain, and bilateral lower extremity edema. He was born consanguineous and healthy.

His medical history included cataract surgery at the age of four. The patient had Marfanoid features and cognitive impairment (Figures 1A and 1B). On physical examination, rhonchi in the lower zone of the left lung and generalized edema in both the lower extremities were noted. Severe acute respiratory syndrome coronavirus 2 (SARS-CoV-2) polymerase chain reaction (PCR) test results of a nasopharyngeal sample were positive. A chest computed tomography (CT) scan showed a subpleural patchy ground-glass area in the left lower lobe. Doppler venous ultrasonography revealed deep venous thrombosis (DVT) in both lower extremities. A complete blood count revealed lymphopenia, with a count of 700/mm^3^. Prothrombin time/International Normalized Ratio, D-dimer, and fibrinogen levels were 1.35 (0.8-1.2), 19.5 μgFEU/mL (0-0.5 μgFEU/mL), and 699 mg/dL (193-412 mg/dL), respectively. Tests for the thrombophilic state, including factor V Leiden mutation, protein S and antithrombin levels, and antiphospholipid antibody, were normal. The patient was started on anticoagulant treatment with low-molecular-weight heparin. A diagnosis of homocystinuria was considered because of the patient's marfanoid appearance, history of cataract surgery, and presence of venous thrombosis. Serum homocysteine levels were above 257 nmol/mL (0-10 nmol/mL), vitamin B12 levels were 100 pg/mL (197-771 pg/mL), and folate levels were 3.07 ng/mL, hemoglobin was 8.1 gr/dL (10.2 - 13.2 gr/dL). The patient was started on 1 mg hydroxocobalamin and 5 mg folic acid daily. At the follow-up two weeks later, serum folic acid and vitamin B12 levels ​​returned to normal, and serum homocysteine levels increased to 324 nmol/mL. The methionine level in the blood amino acids was 49 µmol/L. The patient was started on 10 mg/kg of pyridoxine to evaluate the pyridoxine response. One week later, serum homocysteine level decreased to 49 nmol/mL. A diagnosis of homocystinuria with B6 response was made. Pyridoxine was administered orally. Low-molecular-weight heparin was then administered. Homocystinuria gene panel testing was performed in a genetic laboratory. Monthly follow-up was planned for clinical evaluation and treatment response. 

**FIGURE 1A:**
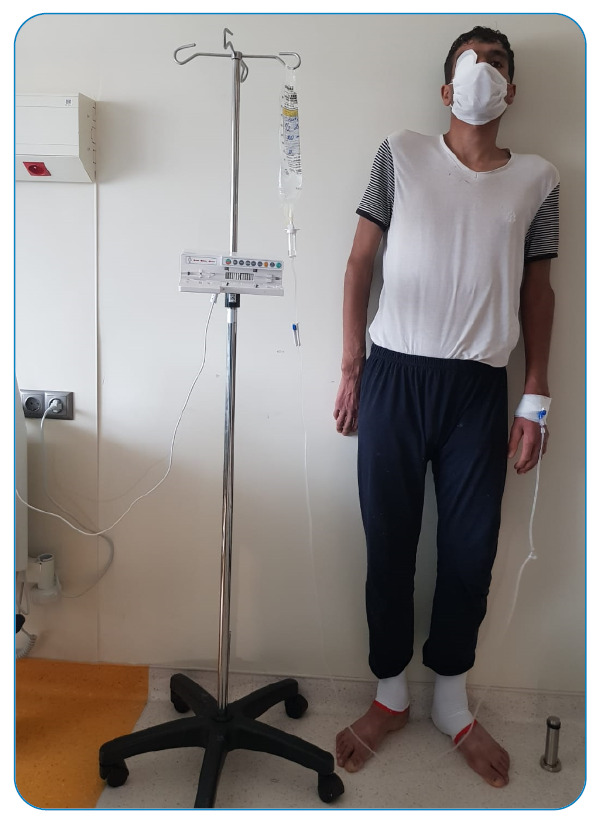
Marfan phenotype of the patient.

**FIGURE 1B:**
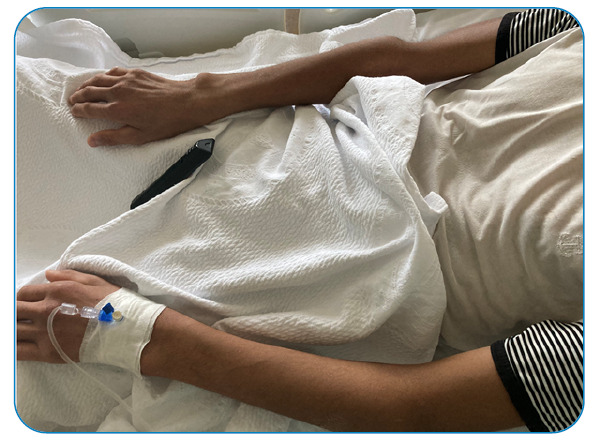
Marfan phenotype of the patient.

## DISCUSSION

Homocystinuria is a treatable autosomal recessive genetic disorder with a prevalence of 1/344000^5^. In this disease, abnormalities in the transsulfuration pathway of methionine metabolism secondary to cystathionine beta-synthase deficiency result in the accumulation of homocysteine, methionine, and their S-adenosyl derivatives in the cells and plasma. The eye, skeleton, central nervous system, and vascular system are the most commonly affected^6^. The most important cause of mortality and morbidity is a thromboembolic complication. The thromboembolic phenomenon can affect both arteries and veins, but the central nervous system, lungs, carotids, and renal arteries have been reported as the most common sites^7^. Al Humaidan et al.^8^ reported a case of intestinal thrombosis with homocystinuria and lower GI bleeding as an outlier. Our patient had venous thrombosis in both lower extremities. Although the clinical manifestations of the disease usually occur in the first year of life, the diagnosis may be delayed, as in our case. Quintas et al.^9^ reported a case of a late diagnosis of homocystinuria presenting with cerebral venous thrombosis. There are also reports of homocystinuria after normal newborn screening^10^. 

Although COVID-19 affects respiratory systems most commonly, it is well known that coagulation function is significantly damaged in COVID-19 patients in both the general ward and intensive care units^11^. Previous studies have reported high rates of venous thromboembolism, up to 25-30%, especially in critically ill patients with COVID-19^3^. In our case, pulmonary embolism and DVT have been reported as the most common thromboembolic events in COVID-19 patients^12^. Although randomized controlled studies are lacking, the current literature shows hyperinflammation caused by SARS-CoV-2 infection, elevated secretion of pro-inflammatory cytokines, and hypercoagulable state caused by disrupted vascular endothelial cells and the anticoagulant system may contribute to the pathophysiology of thromboembolic events^2^ ^,^ ^3^ ^,^ ^11^ ^,^ ^12^. 

Venous thrombosis has many underlying medical conditions and predisposing factors. For example, Homocystinuria is a well-known risk factor for vascular complications.

In our case, the coexistence of COVID-19 with the underlying disease predisposing patients to coagulopathy appeared to contribute to venous thrombosis^2^ ^-^ ^4^. The presence of deep venous thrombosis with further clues such as cataract and marfanoid appearance should lead to a suspicion of homocystinuria, even in late childhood and adolescence. We would like to emphasize the importance of considering homocystinuria in the differential diagnosis to ensure early diagnosis, intervention, and treatment so that the risk of life-threatening complications such as thromboembolic conditions can be reduced and eliminated. 

## INFORMED CONSENT

Informed consent, including the reported image and other clinical information, was obtained from the patient’s parents.

## References

[1] World Health Organization (2022). Coronavirus disease (COVID-19) situation reports.

[2] Connors JM, Levy JH (2020). COVID-19 and its implications for thrombosis and anticoagulation. Blood.

[3] Ali MAM, Spinler SA (2021). COVID-19 and thrombosis: From bench to bedside. Trends Cardiovasc Med.

[4] Caldwell ES (1971). Homocystinuria. JAMA.

[5] Mudd SH, Levy HL, Skovby F, Scriver CR Beau (1995). The Metabolic and Molecular Bases of Inherited Disease.

[6] De Franchis R, Sperandeo MP, Sebastio G, Andria G (1998). Clinical aspects of cystathionine-synthase deficiency: how wide is the spectrum? The Italian Collaborative Study Group on Homocystinuria. Eur J Pediatr.

[7] Generoso A, Fowler B, Sebastio G, Fernandes J, Saudubray J, Berghe G, Walter J (2006). Inborn Metabolic Diseases: Diagnosis and Treatment.

[8] Al Humaidan M, Al Sharkawy I, Al Sanae A, Al Refaee F (2013). Homocystinuria with lower gastrointestinal bleeding: first case report. Med Princ Pract.

[9] Quintas S, Dotor-García Soto J, Alonso-Cerezo MC, Carreras MT (2018). Late diagnosis of homocystinuria in an adult after extensive cerebral venous thrombosis. Pract Neurol.

[10] Asamoah A, Wei S, Jackson KE, Hersh JH, Levy H (2021). Diagnosis of Classic Homocystinuria in Two Boys Presenting with Acute Cerebral Venous Thrombosis and Neurologic Dysfunction after Normal Newborn Screening. Int J Neonatal Screen.

[1] Dobesh PP, Trujillo TC (2020). Coagulopathy, Venous Thromboembolism, and Anticoagulation in Patients with COVID-19. Pharmacotherapy.

[12] Hanff TC, Mohareb AM, Giri J, Cohen JB, Chirinos JA (2020). Thrombosis in COVID-19. Am J Hematol.

